# Expression of FGF23 and α-KLOTHO in Normal Human Kidney Development and Congenital Anomalies of the Kidney and Urinary Tract (CAKUT)

**DOI:** 10.3390/biom15060811

**Published:** 2025-06-04

**Authors:** Patricija Bajt, Anita Racetin, Nela Kelam, Nikola Pavlović, Petar Todorović, Marinela Jelinčić Korčulanin, Natalija Filipović, Ivana Kuzmić Prusac, Fila Raguž, Katarina Vukojević

**Affiliations:** 1 Department of Anatomy, Histology and Embryology, School of Medicine, University of Split, 21000 Split, Croatia; patricija.bajt@mefst.hr (P.B.); anita.racetin@mefst.hr (A.R.); nela.kelam@mefst.hr (N.K.); nikola.pavlovic@mefst.hr (N.P.); petar.todorovic@mefst.hr (P.T.); natalija.filipovic@mefst.hr (N.F.); 2 Department of Pathology, University Hospital Split, Spinciceva 1, 21000 Split, Croatia; ivana.kuzmic-prusac@mefst.hr; 3 Department of Nephrology, University Clinical Hospital Mostar, 88000 Mostar, Bosnia and Herzegovina; fila.raguz@mef.sum.ba; 4 Department of Anatomy, School of Medicine, University of Mostar, 88000 Mostar, Bosnia and Herzegovina; 5 Center for Translational Research in Biomedicine, School of Medicine, University of Split, 21000 Split, Croatia; 6 Mediterranean Institute for Life Sciences, University of Split, Meštrovićevo Šetalište 45, 21000 Split, Croatia

**Keywords:** FGF23, α-KLOTHO, CAKUT, congenital anomalies of the kidney and urinary tract

## Abstract

Congenital anomalies of the kidney and urinary tract (CAKUT) are a major cause of pediatric renal failure, but the molecular mechanisms driving these conditions are not yet fully understood. Fibroblast Growth Factor 23 (FGF23) and its co-receptor α-KLOTHO play crucial roles in regulating calcium and phosphate homeostasis in adult kidneys, but their roles in kidney development and the pathogenesis of CAKUT remain unclear. Because of that, we analyzed the spatial and temporal expression of FGF23 and α-KLOTHO in normal fetal kidney development and CAKUT using an immunofluorescence technique. Our results demonstrate a dynamic pattern of FGF23 and α-KLOTHO expression in healthy kidney development, with FGF23 levels decreasing and α-KLOTHO levels increasing with gestational age. Also, we showed that FGF23 expression was significantly reduced in horseshoe (HKs) and duplex kidneys (DKs), while α-KLOTHO expression remained unchanged across all CAKUT conditions. Based on our results, we suggest that altered FGF23 expression in CAKUT contributes to disease pathogenesis and may represent a potential therapeutic target.

## 1. Introduction

Congenital anomalies of the kidney and urinary tract (CAKUT) are among the most prevalent birth defects globally, contributing significantly to renal failure in pediatric patients [1,2]. These anomalies result from developmental obstructions in the normal kidney and urinary tract during embryogenesis. CAKUT accounts for approximately 23% of all birth defects and is responsible for 40–50% of pediatric end-stage kidney disease cases [3,4]. The spectrum of CAKUT includes a variety of anomalies, which can occur individually or in combination with other syndromes. CAKUT defects can arise due to genetic, epigenetic, or environmental factors [5]. As a major contributor to kidney failure in children, CAKUT is a critical area of focus for early diagnosis and intervention [6]. However, studies on newborns with CAKUT are limited due to challenges in early diagnosis, ethical considerations, and the complexity of genetic analysis in this age group [7]. Since most causes of CAKUT remain unknown, it is crucial to investigate new potential factors, which is why we focused our research on FGF23 and α-KLOTHO.

Fibroblast Growth Factor 23 (FGF23), along with parathyroid hormone and vitamin D, plays an important role in the regulation of calcium and phosphate homeostasis in adults [8]. Secreted mostly by bone, FGF23 primarily acts on the kidneys and parathyroid glands through its interaction with FGF receptor (FGFR) and the co-receptor α-KLOTHO. The presence of α-KLOTHO enhances FGF23 binding to FGFR by 20-fold. However, FGF23 has also been reported to function independently of α-KLOTHO in other organs such as the heart and liver [9]. In the kidneys, FGF23 lowers blood phosphate by reducing its reabsorption in the kidneys and decreasing vitamin D production, which in turn limits phosphate absorption in the intestines. It also promotes calcium and sodium reabsorption [10]. In addition to its function as a co-receptor for FGF23, α-KLOTHO is involved in antioxidative, anti-aging, pro-autophagic, and anti-apoptotic processes [11,12]. Both FGF23 and α-KLOTHO are recognized as early markers of chronic kidney disease (CKD) and its progression, particularly in children with CAKUT. They can help identify the onset and assess the severity of the disease [13]. Despite this, the precise role of these proteins during prenatal development, as well as their potential involvement in the formation of CAKUT, remains unclear.

Since FGF23 and α-KLOTHO play a key role in mineral homeostasis in adult kidneys, it is important to understand their roles during normal kidney development. As these proteins are likely important in kidney development, our study aimed to investigate the expression of FGF23 and α-KLOTHO in both healthy kidney development and in CAKUT. The results could offer valuable insights into kidney development, the causes of CAKUT, and the potential of FGF23 and α-KLOTHO as targets for treatment in kidney diseases.

## 2. Materials and Methods

### 2.1. Kidney Tissue Collection and Preparation

This study used 43 samples of human fetal kidney tissue (Table 1), acquired from spontaneous pregnancy losses and eugenic abortions due to severe abnormalities. The samples were collected at University Hospital Split from the Department of Pathology. The processing of the tissue sections complied with the Declaration of Helsinki and received approval from the Ethical and Drug Committee at the University Hospital in Split (reference: 003-08/23-03/0015, approval number: 2181-198-03-04-23-0073). The gestational age was estimated based on external measurement and menstrual data. Kidney pathology was classified through gross morphological and standard histopathological evaluation.

The kidney tissue was fixed in 4% paraformaldehyde, sectioned into blocks, processed through a graded alcohol series, and subsequently embedded in paraffin. Tissue sections were obtained using cutting blocks with a microtome at a thickness of 5 µm.

### 2.2. Separation of Samples Based on Developmental Phases

We divided our samples based on a previously established classification of developmental phases that focuses on specific developmental structures. According to the classification, Phase 1 occurs from the 5th to the 14th week of development. Phase 2 begins around the 15th week and ends between the 20th and 22nd week. Phase 3 lasts until the 36th week of development, while Phase 4 starts at the 36th week and continues through the postnatal period [14,15,16]. For our analysis, we focused specifically on Phases 2, 3, and 4. Phase 1 was not included in our analysis due to the extreme difficulty in obtaining samples at this early stage of development (Table 1).

### 2.3. Immunofluorescence

Paraffin tissue sections were first deparaffinized in xylol and rehydrated in an alcohol series. Antigen retrieval was achieved by cooking the sections in 0.01 M citrate buffer (pH 6.0). The sections were then washed three times with PBS and treated with a blocking solution (ab64226, Abcam, Cambridge, UK) for 20 min. After removing the blocking solution, primary antibodies were added and incubated in a humidity chamber overnight. Afterwards, the sections were washed in PBS, incubated with secondary antibodies for 1 h at room temperature (RT), and subsequently washed three times in PBS. DAPI (4′,6-diamidino-2-phenylindole), a fluorescent dye used for visualizing nuclei, was applied for 2 min. Finally, tissue sections were covered with coverslips using a mounting medium (Immuno-Mount, Thermo Shandon, Pittsburgh, PA, USA). Samples where the primary antibody was not added were used as negative controls to determine specificity. The antibodies used in this study are listed in Table 2.

### 2.4. Imaging and Quantitative Analysis

Microphotographs of human fetal kidney tissues were captured using an epifluorescence microscope (Olympus BX51, Tokyo, Japan) with a Nikon DS-Ri2 camera (Nikon Corporation, Tokyo, Japan) and NIS-Elements F software (version 5.22.00). The expression of FGF23 and α-KLOTHO was analyzed in 10 separate regions of the healthy fetal kidney cortex and medulla for each sample. Due to the unique structural abnormalities in kidneys affected by CAKUT, distinguishing between the cortex and medulla was challenging in certain phenotypes, particularly in dysplastic kidneys (DYSs). To mitigate this limitation, we analyzed at least 20 visual fields in the group of kidneys affected by CAKUT. Images were captured at 40× magnification with standardized exposure times. Positive staining for FGF23 and α-KLOTHO was observed as diffuse or punctate green fluorescence. The captured images were analyzed using ImageJ software (version 1.54, National Institutes of Health, Bethesda, MD, USA), as described in previous publications [16,17]. Fluorescence overlap was minimized by subtracting the red channel from the green fluorescence. A median filter with a 5.0-pixel radius was applied to duplicated images, and positive signals were extracted by subtracting the filtered images from the originals. Processed images were converted to 8-bit images and modified using the triangle thresholding algorithm. The fluorescence area percentage was then calculated using the “Analyze Particles” tool. To address observer variability, three expert histologists independently analyzed the microphotographs and set thresholds using negative control images. Consistency was confirmed with an intraclass correlation coefficient above 0.8, indicating excellent agreement [18].

### 2.5. Statistical Analysis

Statistical analyses were conducted with GraphPad Prism v8.4.3 (GraphPad Software, La Jolla, CA, USA), with significance set at *p* < 0.05. The normality of the data distribution was assessed using the Shapiro–Wilk test. FGF23 and α-KLOTHO expression levels in the cortex and medulla of healthy fetal kidneys during developmental Phases 2, 3, and 4 were analyzed using one-way ANOVA, followed by Tukey’s post hoc test or the Kruskal–Wallis test, followed by Dunn’s post hoc analysis. In addition, their expression levels in healthy fetal kidneys were compared to those in CAKUT (duplex kidneys (DKs), horseshoe kidneys (HKs), hypoplastic (HYPs), and dysplastic kidneys (DYSs)) using the Kruskal–Wallis test, followed by Dunn’s post hoc analysis. Graphs were created using GraphPad Prism 8.4.3, while the figures were processed with Adobe Photoshop software v9.0. Microphotographs underwent background subtraction and contrast enhancement for improved presentation.

## 3. Results

### 3.1. FGF23 Expression

**Figure 1:**
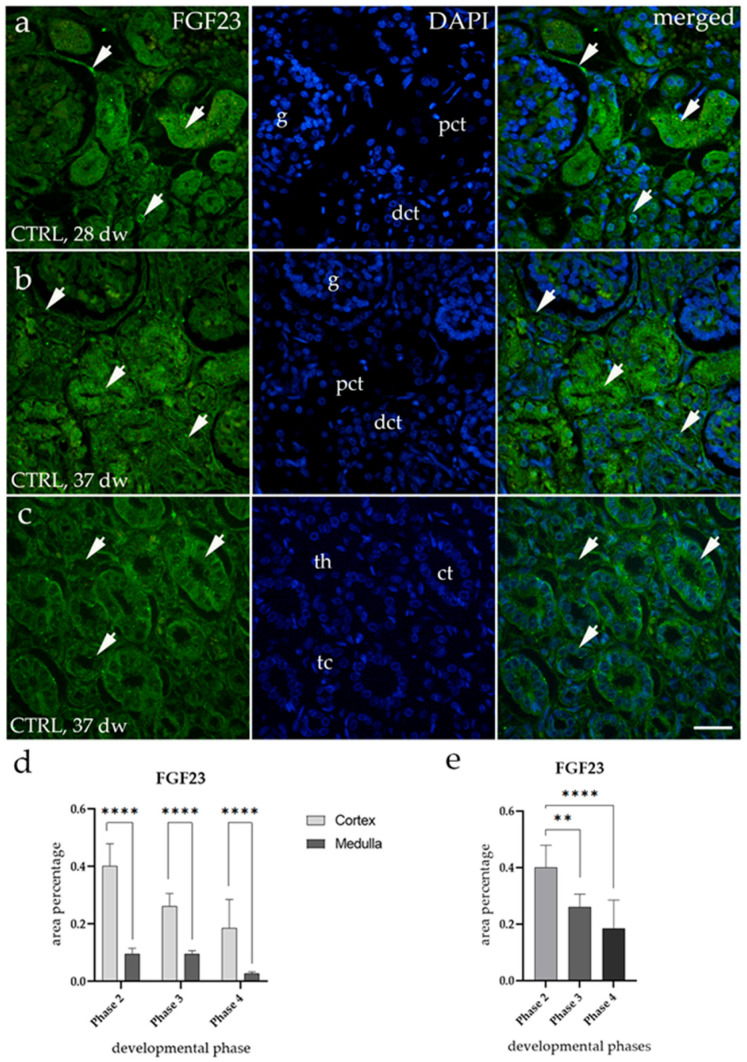
Immunofluorescence staining of the renal cortex (**a**,**b**) and medulla (**c**) of control human fetal kidneys (CTRLs) at the 28th and 37th developmental week (dw), using an antibody for fibroblast growth factor-23 (FGF23). Arrows indicate the localization of FGF23 in the parietal layer of Bowman’s capsule (g), proximal convoluted tubules (pcts), and distal convoluted tubules (dcts) of the renal cortex (**a**,**b**), as well as in the collecting tubules (cts) and the thick (tc) and thin (th) segments of the loop of Henle of the renal medulla (**c**), as marked in the 4′,6-diamidino-2-phenylindole (DAPI)-stained images. Images show separate staining of FGF23, DAPI, and their merged views of fetal kidneys. All images were taken at 40× magnification with a scale bar of 60 µm. The histogram of area percentages of FGF23 in CTRL tissues across developmental Phases 2, 3, and 4 in the cortex and medulla (**d**). Data are presented as the mean ± standard deviation (SD) and were analyzed using a one-way ANOVA, followed by Tukey’s post hoc test for multiple comparisons. Statistically significant results are indicated by **** *p* < 0.0001. The histogram illustrates the area percentage of FGF23, specifically in the cortex during Phases 2, 3, and 4 (**e**). Data are presented as the mean ± standard deviation (SD) and were analyzed using a one-way ANOVA, followed by Tukey’s post hoc test for multiple comparisons. Statistically significant results are marked by ** *p* < 0.01 and **** *p* < 0.0001. Ten representative images of the medulla and cortex were analyzed for each sample.

In the cortex of control fetal kidneys (CTRLs), FGF23 exhibited a diffuse green fluorescence signal in the parietal layer of Bowman’s capsule and the distal convoluted tubules, while a lower signal was observed in the proximal convoluted tubules (Figure 1a,b). In the medulla, FGF23 was expressed as a punctate signal in the collecting ducts and the thick segment of the loop of Henle, whereas very low expression was detected in the thin segment of the loop of Henle (Figure 1c). Quantitative analysis revealed that, across all observed developmental phases, the percentage of FGF23-positive cells was significantly higher in the cortex compared to the medulla (**** *p* < 0.0001, Figure 1d). Furthermore, FGF23 expression in the cortex of healthy kidneys showed a significant decline during fetal aging (** *p* < 0.01, **** *p* < 0.0001, Figure 1e). This decrease in fluorescence intensity is shown in the 28th gestational week (Phase 3) compared to the 37th gestational week (Phase 4) (Figure 1a,b).

### 3.2. FGF23 Expression Is Downregulated in HKs and DKs

**Figure 2:**
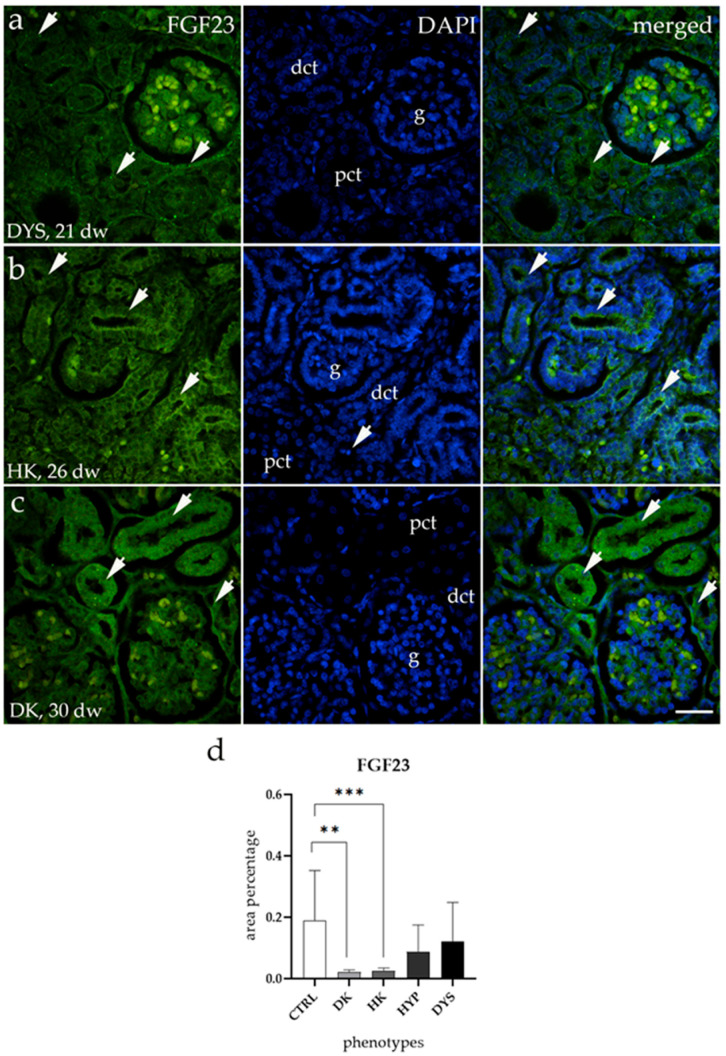
Immunofluorescence staining of human fetal kidneys using an FGF23 antibody (**a**–**c**). Arrows highlight the localization of FGF23 in dysplastic kidneys (DYSs), horseshoe kidneys (HKs) and duplex kidneys (DKs), as marked in the 4′,6-diamidino-2-phenylindole (DAPI) image. Images show separate staining of FGF23, DAPI, and their merged views for DYSs at the 21st developmental week (dw) (Phase 2) (**a**), HKs at the 26th dw (Phase 3) (**b**), and DKs at the 30th dw (Phase 3) (**c**). All images were taken at 40× magnification with a scale bar of 60 µm. The histogram of the area percentages of FGF23 in CTRL, DK, HK, HYP, and DYS fetal kidney tissues (**d**). Data are represented as the mean ± SD (vertical line) and analyzed using an Kruskal–Wallis test followed by Dunn’s multiple pairwise comparisons test. Statistically significant differences are marked by ** *p* < 0.01 and *** *p* < 0.001. Twenty representative images were analyzed for each sample.

FGF23 staining patterns in hypoplastic (HYPs) and dysplastic kidneys (DYSs) were similar to those in CTRL kidneys (Figure 2a). In contrast, horseshoe kidneys (HKs) and duplex kidneys (DKs) showed much lower FGF23 expression, mainly in the parietal layer of Bowman’s capsule and the proximal and distal tubules (Figure 2b,c). A comparison of the area percentage of FGF23 expression between CTRL and CAKUT showed significantly lower expression in DK and HK (** *p* < 0.01 and *** *p* < 0.001, Figure 2d). However, HYPs and DYSs showed no significant difference in expression compared to CTRLs.

### 3.3. α-KLOTHO Expression

**Figure 3:**
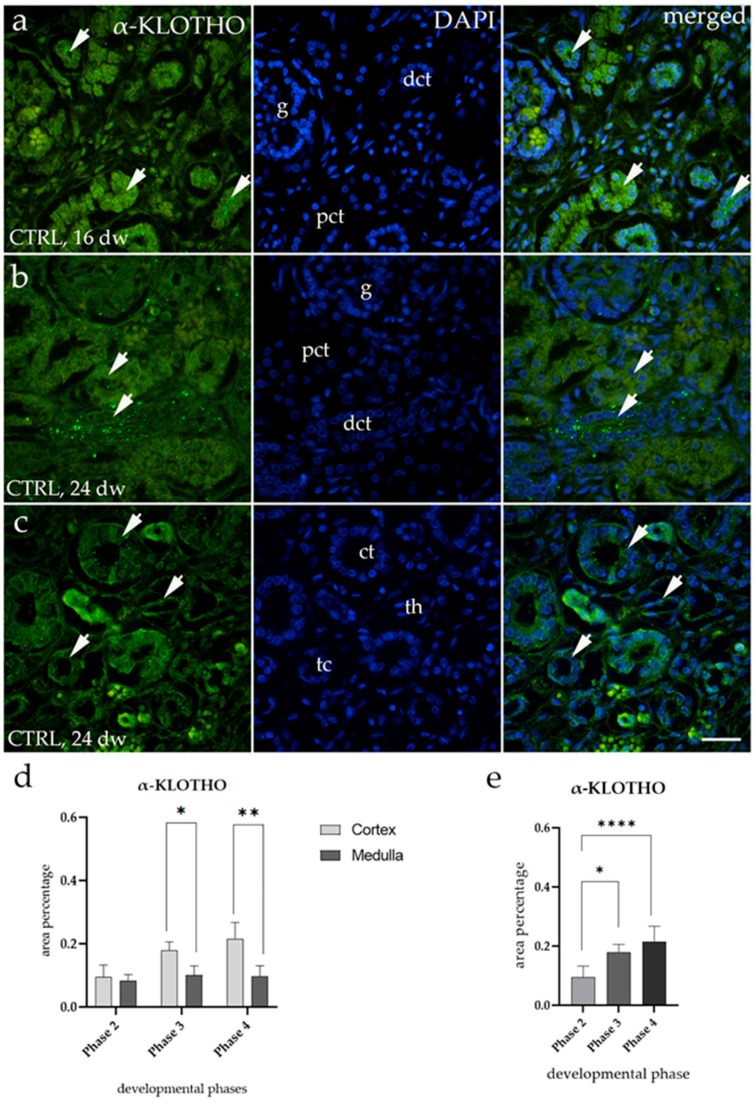
Immunofluorescence staining of the renal cortex (**a**,**b**) and medulla (**c**) of control human fetal kidneys (CTRLs) at the 16th and 24th developmental week (dw), using an antibody for α-KLOTHO. Arrows indicate the localization of α-KLOTHO in the proximal convoluted tubules (pcts) and distal convoluted tubules (dcts) of the renal cortex (**a**,**b**), as well as in the collecting tubules (cts) and the thick (tc) and thin (th) segments of the loop of Henle of the renal medulla (**c**), as marked in the 4′,6-diamidino-2-phenylindole (DAPI)-stained images. Images show separate staining of α-KLOTHO, DAPI, and their merged views of fetal kidneys. All images were taken at 40× magnification with a scale bar of 60 µm. The histogram of area percentages of α-KLOTHO in CTRL tissues across developmental Phases 2, 3, and 4 in the cortex and medulla (**d**). Data are presented as the mean ± standard deviation (SD) and were analyzed using an Kruskal–Wallis test followed by Dunn’s multiple pairwise comparisons test. Statistically significant results are indicated by * *p* < 0.05, ** *p* < 0.01. The histogram illustrates the area percentage of α-KLOTHO specifically in the cortex during Phases 2, 3, and 4 (**e**). Data are presented as the mean ± standard deviation (SD) and were analyzed using an Kruskal–Wallis test followed by Dunn’s multiple pairwise comparisons test. Statistically significant results are marked by * *p* < 0.05 and **** *p* < 0.0001. Ten representative images of medulla and cortex were analyzed for each sample.

The spatial distribution of α-KLOTHO in the cortex of CTRLs showed higher expression in the distal convoluted tubules, with lower expression in the proximal convoluted tubules (Figure 3a,b). In the medulla, α-KLOTHO was localized as a punctate signal in the collecting ducts and the thick segment of the loop of Henle, while minimal expression was observed in the thin segment of the loop of Henle (Figure 3c). Quantitative analysis revealed that the percentage of α-KLOTHO-positive cells was significantly higher in the cortex than in the medulla during Phase 3 and Phase 4 compared to Phase 2 (* *p* < 0.05, ** *p* < 0.01, Figure 3d). Moreover, α-KLOTHO expression in the cortex of healthy kidneys showed a significant increase with fetal aging (* *p* < 0.05, **** *p* < 0.0001, Figure 3e). This increase in fluorescence intensity is evident when comparing the 16th gestational week (Phase 3) with the 24th gestational week (Phase 4) (Figure 3a,b).

### 3.4. α-KLOTHO Expression Remains Consistent Across All Tested CAKUT Conditions

**Figure 4:**
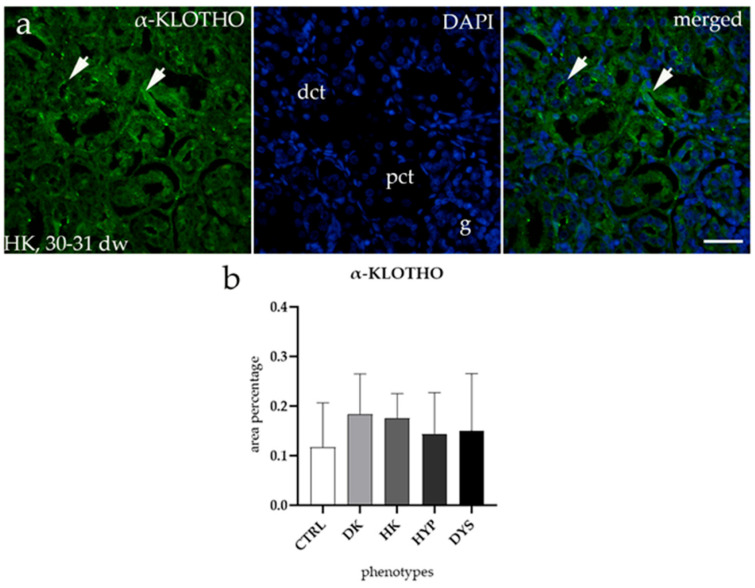
Immunofluorescence staining of human fetal kidneys using α-KLOTHO antibody (**a**). Arrows highlight the localization of α-KLOTHO in horseshoe kidneys (HKs), as marked in the DAPI image. Image shows separate staining of α-KLOTHO, DAPI, and their merged views for HKs at the 30–31st developmental week (Phase 3). Image was taken at 40× magnification with a scale bar of 60 µm. The histogram of the area percentages of α-KLOTHO in CTRL, DK, HK, HYP and DYS fetal kidney tissues (**b**). Data are represented as the mean ± SD (vertical line) and analyzed using an Kruskal–Wallis test followed by Dunn’s multiple pairwise comparisons test. Twenty representative images were analyzed for each time point.

α-KLOTHO staining patterns in all observed phenotypes were similar to those observed in CTRL kidneys (Figure 4a), and therefore only expression in HKs is shown. The expression of α-KLOTHO was higher in the distal convoluted tubules and lower in the proximal convoluted tubules. When comparing the area percentage of expression between CTRL and CAKUT samples, no significant differences were found (Figure 4b).

## 4. Discussion

In this study, we analyzed the expression patterns of FGF23 and α-KLOTHO during normal fetal kidney development and CAKUT. We demonstrated that FGF23 expression is altered, while α-KLOTHO expression remains unchanged in CAKUT. These findings suggest a potential role for FGF23 in the pathogenesis of these anomalies, whereas the stable expression of α-KLOTHO suggests that it may play a less significant role in CAKUT. In healthy fetal kidneys, we found that FGF23 expression decreases, while α-KLOTHO increases with gestational age, suggesting their dynamic roles in kidney tissue maturation.

Our study showed that FGF23 expression was significantly lower in horseshoe (HKs) and duplex kidneys (DKs) compared to control samples, indicating its potential role in abnormal kidney morphogenesis. Previous studies have shown that FGF23 is crucial for mineral homeostasis in postnatal life, but plays a minimal role during fetal development due to the placental regulation of phosphate metabolism [19,20]. This finding suggests that FGF23 may have other important roles during development, potentially including a role in kidney development. Zhao et al. demonstrated that decreased FGF23 inhibits placental angiogenesis through the MAPK/ERK signaling pathway in preeclampsia [21]. This suggests that FGF23 may also play a crucial role in kidney vascular development during pregnancy. Additionally, the MAPK/ERK signaling pathway is known to play a crucial role in kidney development, regulating ureteric bud branching morphogenesis and maintaining the stability of the metanephric blastema [22]. Based on these findings, lower FGF23 levels may impair angiogenesis via the MAPK/ERK pathway within the fetal kidney, leading to insufficient vascular support and potentially disrupting proper branching and the stability of the metanephric blastema. This disruption may lead to abnormal ureteric bud branching and the development of a DK [23], as well as the fusion of the mesenchymal blastema, resulting in a HK [24]. This aligns with studies indicating that altered MAPK/ERK signaling is involved in CAKUT formation, with alterations caused by gestational diabetes and *Gen1* and *Robo2*, which affect urinary tract development [25,26]. While these studies involve different ligands, they highlight the critical role of MAPK/ERK signaling in kidney morphogenesis. As FGF23 also modulates this pathway, its altered expression may contribute to CAKUT. Further studies are needed to better understand the relationship between FGF23 and the MAPK/ERK pathway in kidney development and CAKUT disorders.

In our study, α-KLOTHO expression remained unchanged across all CAKUT conditions, while FGF23 expression was altered. This difference may be explained by potential disruptions in the posttranslational modifications of FGF23, while α-KLOTHO remains unaltered. It is known that FGF23 activation requires both FGFR and α-KLOTHO. In normal tissues, furin plays a critical role in regulating FGF23 levels by mediating its cleavage, generating C- and N-terminal fragments that separate the binding domains for FGFR and α-KLOTHO [27,28,29]. This highlights the importance of the precise regulation of FGF23′s post-translational modifications, as alterations at modification sites or disruptions in its processing can either inhibit or enhance FGF23 cleavage [27]. Therefore, it is possible that, while α-KLOTHO expression remains unchanged, disruptions in post-translational modifications could reduce FGF23 levels, potentially contributing to the development of CAKUT. However, several studies have suggested that α-KLOTHO may play a role in kidney development. For example, α-KLOTHO supplementation has been shown to improve kidney function and reduce damage in neonatal models [11], while other studies suggest that it supports glomerular and tubular development through VEGF-driven kidney formation [12]. While these findings highlight α-KLOTHO’s potential role in kidney function and vascular stability, our results suggest that it may not be directly involved in CAKUT pathogenesis. However, further studies are needed to clarify its precise role in congenital kidney anomalies.

Our study revealed dynamic changes in FGF23 and α-KLOTHO expression throughout fetal kidney development. We observed a gradual decrease in FGF23 expression over time, with higher levels in the cortex than in the medulla at all developmental stages. This decrease may be explained by the transition of phosphorus regulation from the placenta to the kidneys. As fetal development progresses, the placenta gradually reduces its role in regulating phosphorus balance. The kidneys then take over this function, which may lead to the stabilization of FGF23 levels at postnatal levels [19]. Furthermore, prior studies on zebrafish by Mangos et al. were the only ones to describe the spatial and temporal expression of FGF23 in embryonic tissues and adult kidneys. Their findings indicate that FGF23 appears after organogenesis and remains present in adulthood in the corpuscles of Stannius, near the nephron, where it contributes to calcium and phosphate balance [30]. In contrast, our study showed that α-KLOTHO expression increases as kidney development progresses. This aligns with the study in mice by Song et al., who also demonstrated a temporal increase in α-KLOTHO expression, showing that it rises from embryonic day 16 to postnatal day 1. They also found that this increase may be linked to tubular cell proliferation, as α-KLOTHO-positive tubular cells colocalize with proliferation markers [31]. This could explain why α-KLOTHO expression rises during fetal development. Furthermore, we showed that α-KLOTHO is initially present in both the cortex and medulla, but that its expression becomes stronger in the cortex as development progresses, which aligns with findings in fetal mouse kidneys [31]. In adult human kidneys, α-KLOTHO is expressed only in the cortex, suggesting that its expression in the medulla may disappear during development [32]. Additionally, a study on zebrafish also detected α-KLOTHO during the embryonic period in the distal pronephros, brain, and pancreas [30]. Finally, our observations indicate that FGF23 and α-KLOTHO colocalize in both the cortex and medulla, suggesting a potential functional interaction in renal development.

Our study has several limitations that should be acknowledged. The primary limitation is the small sample size for each CAKUT subtype, along with the observational nature of our research. Although the sample size is small, the study is based on highly valuable human material that is very difficult to obtain, making the insights gained from this research of immeasurable importance. Furthermore, the analyzed samples consist of archived, formalin-fixed, and paraffin-embedded human fetal tissue, which restricted the use of quantitative protein analysis methods such as flow cytometry and Western blotting. Formalin fixation can cross-link proteins, which may potentially alter their structure and prevent the binding of antibodies, which is essential for both Western blotting and flow cytometry. Additionally, the absence of samples from Phase 1 of fetal kidney development limits the scope of our analysis and the strength of potential conclusions.

## 5. Conclusions

This study provides valuable insights into the expression patterns of FGF23 and α-KLOTHO during fetal kidney development and their potential roles in CAKUT. Our results show that FGF23 expression is significantly altered in specific CAKUT subtypes, such as HKs and DKs, suggesting that disruptions in FGF23 may contribute to abnormal kidney development. In contrast, α-KLOTHO expression remained consistent across all CAKUT conditions, implying that its role in these anomalies may be indirect. Our findings also highlight the changes in FGF23 and α-KLOTHO expression during fetal kidney development, with FGF23 decreasing and α-KLOTHO increasing, especially in the renal cortex. The interaction between these two proteins should be explored further, as it may be important for understanding kidney development and the development of CAKUT.

## Figures and Tables

**Table 1:** Samples of human fetal kidneys (*n* = 43) analyzed in the study.

Groups	Developmental Phase	Renal and Associated Pathology	Gestational Weeks	Number of Kidney Samples
Healthy kidneys (CTRLs)	Phase 2	N/A *	15	1
		N/A	15–16	2
		N/A	16	1
		N/A	16–17	1
		N/A	18	1
		N/A	18	2
		N/A	21	2
	Phase 3	N/A	24	2
		N/A	28	2
		N/A	28	1
		N/A	32	2
		N/A	35	2
	Phase 4	N/A	37	2
		N/A	38	1
Duplex kidneys (DKs)	Ureter duplex lateris dextri		24	1
	Ureter duplex sinister		30	1
Horseshoe kidneys (HKs)	Ren concreatus arcuatus, cystae multiplices corticales		22	1
	Ren concretus arcuatus, tetralogija Fallot		26	1
	Syndroma Edwards, Ren arcuatus		30–31	1
	Syndroma Edwards, Ren arcuatus		34	1
Hypoplastic kidneys (HYPs)	Hypoplasio renis		28	1
	Hypoplasia renis lateris dextri		30	1
	Hypoplasio renis sinister		37	1
Dysplastic kidneys (DYSs)	Megaureter lateris dextri, dysplasia renalis		21	1
	Agenesia renis dextri, ren unilateralis		21	2
	Ren sinister cysticus, ren dexter absens		21–22	2
	Dysplasia mulicystica renis dextri/Cystes parvae focales		27	2
	Dysplasia Hypoplastica		33–34	1
	Renes dysplastici cystic, Potter syndroma		35	2
	Agenesis renis dextri et dysplasia renis sinistri cum ureter duplex, Curranno sindrom		37	1
	Dysplasia Hypoplastica, renalis bilateralis Down, Potter syndrome		38	1

* N/A—not applicable.

**Table 2:** Primary and secondary antibodies.

Antibodies		Catalog Number	Host	Source	Dilution
Primary	Anti-FGF23 antibody	AF2604	Goat	R&D Systems (Minneapolis, MN, USA)	1:200
	Anti- α-KLOTHO antibody	28100-1-AP	Rabbit	Proteintech Group (Planegg-Martinsried, Germany)	1:200
Secondary	Anti-Goat IgG (H + L), Alexa Fluor^®^ 488	705-545-003	Donkey	Jackson Immuno Research Laboratories, Inc. (Baltimore, PA, USA)	1:300
	Anti-Rabbit lgG (H + L) Alexa Fluor^®^488	711-545-152	Donkey	Jackson Immuno Research Laboratories, Inc., (Baltimore, PA, USA)	1:300

## Data Availability

The original contributions presented in this study are included in the article. Further inquiries can be directed to the corresponding author.
